# Norwegian farmers' vigilance in reporting sheep showing scrapie-associated signs

**DOI:** 10.1186/1746-6148-3-34

**Published:** 2007-12-12

**Authors:** Petter Hopp, Synnøve Vatn, Jorun Jarp

**Affiliations:** 1 Section of epidemiology, National Veterinary Institute Oslo, P.O. Box 8156 Dep., NO-0033 OSLO, Norway; 2 The Norwegian Sheep Health Service, P.O. Box 396 Økern, NO-0513 OSLO, Norway

## Abstract

**Background:**

Scrapie is a chronic neurodegenerative disease affecting small ruminants and belongs to the transmissible spongiform encephalopathies. Scrapie is considered a serious animal disease and it has been notifiable in Norway since 1965. The clinical signs of scrapie might be vague and the farmers, if familiar with the signs of scrapie, are often in the best position for detecting scrapie suspects. In 2002, an anonymous questionnaire survey was conducted in order to assess Norwegian sheep farmers' vigilance of scrapie.

**Results:**

Although the potential detection of a scrapie-positive animal would lead to the destruction of the sheep flock concerned, almost all the farmers (97 %) expressed their willingness to report scrapie suspects. This was most certainly dependent on the Government taking the economic responsibility for the control programme as nearly all the farmers responded that this was an important condition. Listeriosis is relatively common disease in Norwegian sheep and a differential diagnosis for scrapie. In a multinomial logistic regression the reporting behaviour for non-recovering listeriosis cases, used as a measurement of willingness to report scrapie, was examined. The reporting of non-recovering listeriosis cases increased as the knowledge of scrapie-associated signs increased, and the reporting behaviour was dependent on both economic and non-economic values.

**Conclusion:**

The results indicate that in 2002 almost all sheep farmers showed willingness to report any scrapie suspects. Nevertheless there is an underreporting of scrapie suspects and the farmers' awareness and hence their vigilance of scrapie could be improved. Furthermore, the results suggest that to ensure the farmers' compliance to control programmes for serious infectious diseases, the farmers' concerns of non-economic as well as economic values should be considered.

## Background

Scrapie is a fatal neurodegenerative disease affecting sheep and goat. It belongs to the transmissible spongiform encephalopathies (TSE), and there are at least two types: classical and atypical scrapie [1]. From the 1960s, the Norwegian Government has considered scrapie a serious animal disease, and if it was detected attempts would be made to eradicate it [2]. During recent years there has been growing international concern about TSEs in sheep as sheep might have acquired bovine spongiform encephalopathy (BSE) from concentrated feeds contaminated with the BSE agent [3], and BSE and scrapie cannot be differentiated by clinical signs or histopathological findings in the sheep [4]. This has led to the implementation of extensive surveillance programmes and strict control measures for TSEs in small ruminants in several countries [5].

The first case of scrapie in indigenous Norwegian sheep was detected in 1981 and an increasing number of cases was detected in the 1990s [6] leading to the introduction of the Norwegian scrapie surveillance and control programme in 1997. The programme comprises an information campaign aimed at Norwegian sheep farmers, a surveillance programme, control measures in the flocks where scrapie-positive animals have been detected (hereafter referred to as scrapie-positive flocks), and measures at the national level restricting the movement of sheep [7].

From the first scrapie case in 1981, all small ruminants in scrapie-positive flocks were culled [7]. The additional control measures at the flock level included extensive sanitation measures (removal of manure, removal of material unsuited for disinfection, cleaning and disinfection of buildings, changing of upper layer on surrounding roads, ploughing and/or burning of grazing areas and fitting of new fences where in contact with sheep), an empty period of two (three since 2005) years, and the flock being subject to restrictions for five years after restocking. These control measures may also be implemented in flocks that have had contact with scrapie-positive flocks by movement of animals. Until 2004, the control measures applied were equal regardless of whether classical or atypical scrapie was diagnosed. Thereafter, partial depopulation has been used in flocks where atypical scrapie has been found. The farmers receive full compensation for the costs of the animals and the control measures [8]. More than 60 scrapie-positive flocks of totally approximately 20,000 flocks in Norway had been destroyed by the start of this study in 2002. In addition between 600 and 700 contact flocks were depopulated in 1996 to curb the trend of increasing numbers of scrapie-positive flocks [7]. The magnitude of the measures applied to control scrapie surpasses the control measures applied for any other disease experienced by the Norwegian sheep industry at least since the 1950s [9]. We therefore expect the sheep farmers to be especially concerned about the detection of scrapie.

Scrapie has been notifiable in Norway since 1965 [2] and the detection of scrapie was first based on passive surveillance only. In 1997 an active surveillance programme of slaughtered animals was introduced, and in 2002 surveillance of slaughtered animals and fallen stock was implemented in accordance with the Commission Regulation (EC) 999/2001 [5]. In1997, all farmers were given written information that animals showing typical signs of scrapie such as pruritus and neurological signs should be reported to the District Veterinary Officer (DVO) [10] and thereafter all farms have been visited and given information on scrapie by the DVO every second or third year as a part of the scrapie surveillance and control programme. In the early clinical stage, the signs are often vague, such as a change of behaviour, and typical clinical signs may not be seen until late in the incubation period [11] which might last for several years [4]. Important differential diagnoses of scrapie are other diseases that produce neurological signs, including listeriosis [12]. Hence, cases that show signs of listeriosis and do not recover after treatment should be regarded as scrapie suspects.

If the farmer is familiar with the signs of scrapie, he is in the best position to detect scrapie suspects. However, whether the farmer decides to report suspicious clinical cases and found-dead animals or to conceal them will depend on his motivation to report the disease [13]. Therefore, the reporting of scrapie suspects by the farmers is dependent on their knowledge and awareness of the disease and their willingness to report the disease which might be summarised as their vigilance towards the disease [14].

Acknowledging the importance of the farmers for the performance of the scrapie surveillance programmes, a questionnaire survey among Norwegian sheep farmers was conducted in 2002. The aims of the study were to analyse the sheep farmers' vigilance in reporting animals with a higher risk of having scrapie, measured as reporting animals showing typical scrapie-associated signs and non-recovering cases showing signs of listeriosis. In this study no differentiation was made between classical scrapie and atypical scrapie as the same control measures were applied to the two scrapie types in 2002.

## Results

### Response rate

The questionnaires were returned from 2125 of 3000 farmers. Of these, 55 farmers who reported having less than ten breeding sheep were excluded from the study, giving a response rate of 70%. Farmers were considered as drop-outs when they had given no information on region, flock size, scrapie status, and reporting behaviour of non-recovering listeriosis cases or if they gave no answer to any of the questions concerning their knowledge or their willingness to report. In total 97 questionnaires were drop-outs, giving information from 1973 questionnaires (67%) that were available for analysis.

### Description of the study population

#### Farmers' willingness to report scrapie suspects

Most farmers (97%) answered that they would be willing to report scrapie suspects. The remainder constituted those who would slaughter or kill the animal (2%) and those who did not know how they would act in the situation (1%). When there was a non-recovering listeriosis case, 21% of the farmers would report the animal as a scrapie suspect and 51% of the farmers would have the animal re-examined by a veterinary practitioner. The remaining farmers would cull the animal.

#### Farmers' knowledge of scrapie-associated signs

Each of the scrapie-associated signs listed were correctly checked by 34% to 69% of the farmers. Itching and trembling were the most well-known signs, being recognised by 69% and 67% of the farmers, respectively. At least two associated and zero non-associated signs were checked by 71% of the farmers, and at least three associated and zero non-associated signs were checked by 47% of the farmers. Each of the signs considered as not being associated with scrapie was checked by 0.5% to 4% of the farmers. Approximately 2% of the farmers were not familiar with any scrapie-related symptoms, and approximately 6% did not answer the question.

#### Farmers' anticipated reactions if scrapie were to be detected in their flock

In the hypothetical situation where scrapie was detected in one of the farmer's sheep, the statement "Satisfied that the detection of scrapie would enable the eradication of the disease from the flock" was considered to be very important by 55% of the farmers and important or very important by 81% of them (Table 1).

**Table 1:** The Norwegian farmers' responses to a questionnaire study in 2002

		**Reporting behaviour**		
		
		**No. of responses (row %)**		
		
**Explanatory variable/Category**	**N for variable**	**Not report**	**Re-examine**	**Notifying**
Region	1973			
Northern Norway		76 (31)	133 (53)	40 (16)
Middle Norway		102 (32)	154 (48)	64 (20)
Western Norway		211 (25)	448 (53)	190 (22)
South-Eastern Norway		167 (30)	276 (50)	112 (20)
Flock size	1973			
≥ 100 breeding sheep		111 (40)	119 (43)	47 (17)
50–99 breeding sheep		209 (32)	330 (50)	122 (18)
10–49 breeding sheep		236 (23)	562 (54)	237 (23)
Knowledge of scrapie-associated symptoms	1852			
0 signs recognised		13 (37)	22 (63)	0 (0)
1 signs recognised		105 (36)	138 (47)	49 (17)
2 signs recognised		147 (29)	248 (49)	115 (23)
3 signs recognised		143 (27)	290 (55)	92 (18)
4 signs recognised		87 (27)	157 (48)	83 (25)
5 signs recognised		24 (15)	91 (56)	48 (29)
I need more knowledge of scrapie symptoms	1697			
Very important		185 (27)	354 (51)	157 (23)
Less important ^†^		297 (30)	511 (51)	193 (19)
Having easy access to a District Veterinary Officer (DVO)	1856			
Very important		236 (24)	523 (52)	244 (24)
Less important ^†^		286 (34)	418 (49)	149 (17)
Being offered free examination of scrapie suspects	1748			
Very important		244 (29)	376 (45)	213 (26)
Less important ^†^		260 (28)	501 (55)	154 (17)
The Government compensates for the cost of the control measures when scrapie is detected	1815			
Very important		404 (29)	692 (49)	310 (22)
Less important ^†^		111 (27)	221 (54)	77 (19)
Worried about losing income	1737			
Very important		279 (30)	447 (48)	215 (23)
Less important ^†^		222 (28)	424 (53)	150 (19)
Worried about losing work	1622			
Very important and Important		200 (29)	339 (49)	153 (22)
Of minor Importance and Not important		267 (29)	483 (52)	180 (19)
Worried about loss of breeding material	1683			
Very important		188 (27)	350 (51)	147 (21)
Less important ^†^		296 (30)	506 (51)	196 (20)
Worried about the emotional distress of losing animals	1720			
Very important		207 (25)	440 (54)	174 (21)
Less important ^†^		288 (32)	433 (48)	178 (20)
Worried about being accused of spreading scrapie	1631			
Very important and Important		278 (26)	551 (52)	222 (21)
Of minor Importance and Not important		189 (33)	276 (48)	115 (20)
Worried about blaming oneself for having got scrapie	1616			
Very important and Important		220 (26)	455 (53)	181 (21)
Of minor Importance and Not important		250 (33)	361 (48)	149 (20)
Satisfied that the detection of scrapie would enable the eradication of the disease from the flock	1711			
Very important		264 (24)	548 (50)	276 (25)
Less important ^†^		212 (34)	327 (52)	84 (13)

The distribution of the responses is given with regard to the reporting behaviour for non-recovering listeriosis cases.

^† ^Less important includes the categories: Important, Of minor importance, and Not important.

If scrapie should be detected in the farmer's flock, the concerns "Worried about losing income", "Worried about the emotional distress of losing the animals", and "Worried about loss of breeding material" were expected to be very important for 48%, 42% and 35% of the farmers respectively (Table 1). The concern "Worried about being accused of spreading scrapie" was expected to be considered very important by 26% of the farmers. For both the concerns "Worried about blaming oneself for having got scrapie" and "Worried about losing work", less than 14% of the farmers considered these as very important.

#### The farmers' opinion of factors potentially important for reporting behaviour

The statement that "The Government compensates for the cost of the control measures when scrapie is detected" was considered to be very important by 71% of the farmers and important or very important by 87% of the farmers. "Having easy access to a DVO" and "Being offered free examination of scrapie suspects" were considered important or very important by 88% and 70% of the farmers respectively (Table 1).

### Multivariate analysis

In the multivariate analysis (Table 2), the relative risk ratio (RRR) for "Notifying" or "Re-examine" as compared to "Not report" the non-recovering listeriosis cases increased significantly as the farmers' knowledge of scrapie-associated signs increased. For the farmers who would find it very important to be offered free examination of the animal, the RRR for "Notifying" were significantly higher compared to those who would "Re-examine" the animal (RRR = 1.6). The RRR for "Notifying" the non-recovering listeriosis cases was significantly higher for those who would find it very important that the detection of scrapie would enable eradication of scrapie compared both to those who would "Not report" (RRR = 2.4) and those who would "Re-examine" the animal (RRR = 1.2). For the farmers who were concerned that they would be worried about blaming themselves for having got scrapie, the RRR for "Re-examine" was significantly higher compared to "Not report". The variables "Satisfied that the detection of scrapie would enable the eradication of the disease from the flock" and "Worried about blaming themselves for having got scrapie" significantly modified the effect of each other (in the model), so that the effect of these two variables were antagonistic. The RRR for reporting was significantly higher for flocks of less than 50 sheep compared to larger flocks, while geographical region was not a significant explanation variable in the final model.

**Table 2:** The results from the regression analyses of a questionnaire survey performed among Norwegian sheep farmers.

**Explanatory variable/Category**	**Notifying vs Not report**			**Re-examine vs Not report**			**Notifying vs Re-examine**			**Wald type III p-value**
		
	**Beta**	**RRR**	**RRR 95% CI**	**Beta**	**RRR**	**RRR 95% CI**	**Beta**	**RRR**	**RRR 95% CI**	
*Knowledge of scrapie-associated signs*										< 0.001
Continuous	0.3	1.4 ^†^	1.2 – 1.6	0.2	1.3 ^†^	1.1 – 1.4	0.09	1.1	1.0 – 1.2	
*Being offered free examination of scrapie suspects*										0.003
Very important	0.3	1.3	1.0 – 1.8	-0.2	0.8	0.6 – 1.1	0.5	1.6 ^†^	1.2 – 2.1	
Less important ^‡^		1			1			1		
*That the detection of scrapie would enable eradication of scrapie from the flock*										< 0.001
Very important	0.9	2.4 ^†^	1.7 – 3.4	0.2	1.2	1.0 – 1.6	0.7	2.0 ^†^	1.4 – 2.7	
Less important ^‡^		1			1			1		
*Worried about blaming oneself for having got scrapie for the farmers who considered detection of scrapie *Very important										0.74
Very important and Important	0.2	1.3	0.7 – 2.5	0.5	1.7	1.0 – 2.8	-0.3	0.8	0.4 – 1.4	
Of minor Importance and Not important		1			1			1		
*Worried about blaming oneself for having got scrapie for the farmers who considered detection of scrapie *Less important ^‡^										< 0.001
Very important and Important	0.3	1.4	0.7 – 3.0	0.8	2.3 ^†^	1.3 – 3.9	-0.5	0.6	0.3 – 1.2	
Of minor Importance and Not important		1			1			1		
*Flock size*										< 0.001
≥ 100 breeding sheep	-0.9	0.4 ^†^	0.2 – 0.9	-0.9	0.4 ^†^	0.2 – 0.8	-0.01	1.0	0.5 – 2.1	
50–99 breeding sheep	-0.9	0.4 ^†^	0.2 – 0.9	-0.5	0.6	0.4 – 1.1	-0.4	0.7 ^†^	0.3 – 1.4	
10–49 breeding sheep		1			1			1		

The results from the multinomial logistic regression analyses with the reporting behaviour of non-recovering listeriosis cases as the response variable in a questionnaire survey performed among Norwegian sheep farmers in 2002 (N = 1416). Not report was used as the reference group. The parameter estimates for "Worried about blaming oneself for having got scrapie" were presented for each level of the variable "That the detection of scrapie would enable eradication of scrapie from the flock" due to interaction between the variables. The contrast Notifying vs Re-examine is mathematically redundant with the two first contrasts, but is presented for comparative purposes.

Likelihood ratio (chi-square) = 246.8, degrees of freedom = 254, p-value of the final model = 0.62

^† ^p-value of estimate < 0.05

^‡ ^Less important includes the categories: Important, Of minor importance, and Not important.

## Discussion

It is a great strength for the Norwegian scrapie surveillance and control programme that almost all Norwegian sheep farmers in our study answered that they would be willing to report scrapie suspects in their flock. The results indicate that they support the programme, despite the fact that the consequence of detecting a scrapie-positive animal in their flock would be the destruction of all small ruminants in their flock. This was supported by the result that most of the farmers stated that they would consider it important that the detection of scrapie would enable the eradication of the disease from the flock, indicating that the individual farmer takes responsibility for the sheep industry. The active involvement of the farmers' organisations has been important for the implementation of national disease control programmes – for example the programme for bovine virus diarrhoea in cattle [15] – and the result of this study might be interpreted as support from the individual farmer to such a policy.

The high percentage of the farmers who were willing to report scrapie is most certainly dependent on the Government taking the economic responsibility for the control programme since nearly all the farmers responded that this was an important condition. Although depopulation of scrapie-positive flocks might be a cost-effective control option at the national level, the individual farmer may not be able to manage the economic loss imposed by such a policy alone. In many countries the farmers' economic loss resulting from depopulation is therefore partly or fully compensated by the Government, or by using common funds or other insurance systems. In Norway, a standardised compensation per animal destroyed is offered, and the sanitary measures applied are fully covered by the Government. In addition, production subsidies are given for the empty period (up to three years) at the same level as the year prior to the diagnosis [8]. The economic loss is thus at least partly compensated.

Although the farmers might express their true intention of reporting scrapie suspects, it is difficult to know what their behaviour would be when confronted with the actual situation. Results of surveys performed in the Netherlands and Great Britain have shown an underreporting of scrapie suspects [16-18]. During the period from 2000 to 2002, the number of scrapie suspects annually reported decreased from 47 to 39 [19] and decreased further to 25 in 2006. In the Norwegian sheep population more than 2000 clinical listeriosis cases have been reported annually [20]. Assuming 40% to 50% mortality from listeriosis [21] there should be at least 800 found-dead sheep that have shown signs of neurological disorders. Hence, the low number of submitted scrapie suspects indicates an underreporting of these cases in Norway despite that the farmers have expressed willingness to report them.

The fact that only 21% would notify non-recovering listeriosis cases while almost all would notify suspect scrapie cases supports that there is an underreporting in Norway. One explanation might be that listeriosis is a well known disease and that the farmers don't find it necessary to report it as scrapie suspects. However, the fact that "Notifying" non-recovering listeriosis cases was associated with better knowledge of scrapie-associated signs suggests that underreporting might partly be due to lack of knowledge of which signs should be reported as suspicious of scrapie. Whatever the reason, this does not necessarily represent a deliberate underreporting of scrapie to hide the disease.

This might seem to be in contrast to the fact that 88% of the farmers recognised at least one of the scrapie-associated signs. Furthermore, the fact that each of the scrapie-associated signs were correctly identified by 34% to 69% of the Norwegian farmers, seems comparable with the results of studies from Ireland and the Netherlands where each of the scrapie-associated signs was correctly identified by 25% to 50% of the farmers [16,22] However, scrapie has been detected in less than 0.5% of the sheep flocks in the population, and most of the scrapie-positive flocks have been detected in a limited geographical area [6]. Consequently most farmers have had no practical experience of scrapie, and the typical farmer might not be able to recognise the many vague signs of the disease [11], although in theory he is able to identify at least one typical sign.

To control serious infectious diseases it is important to ensure that the behaviour of the farmers does not undermine the disease control programmes. The results of the multivariate analysis indicate that farmers' attitudes and concerns of non-economic values might be important for their reporting behaviour. Therefore, both economic compensation as well as considering the farmers' non-economic values should be taken into account when designing a control programme. This might be valid for all diseases where extensive control measures are put in force. We are not aware that the importance of non-economic values for the reporting behaviour of serious diseases has been examined, and we would suggest that this is explored further in a sociological context.

The disease produces neurological signs that most farmers are expected to be aware of, and the farmers have been given the information that animals that show neurological disorders should be regarded as scrapie suspects. Consequently we chose to use the reporting behaviour of non-recovering listeriosis cases as a measurement of the farmer's vigilance in reporting animals with signs suspicious of scrapie. However, as listeriosis as such is not a notifiable disease, the reporting behaviour of the farmers reflects both their knowledge of scrapie-associated signs and their willingness to report scrapie. The reporting of non-recovering listeriosis cases must therefore be considered an indirect measure for the farmers' vigilance. Nevertheless, we consider the fact that the factors farmers' knowledge, economy and non-economic values were important for the reporting behaviour to be reasonable for non-recovering listeriosis cases, as well as for scrapie suspects.

In accordance with previous questionnaire surveys concerning scrapie [16-18,23] the questionnaire survey was performed as an anonymous study to reduce the potential problem of farmers concealing their true opinion. Although the problem of misleading answers cannot be totally avoided through anonymity, the results of the study indicate that when considering a hypothetical situation most of the respondents were willing to report scrapie suspects in 2002.

The response rate in this study, 70%, is high compared with the typical anonymous postal survey within health sciences [24] as well as other scrapie studies using anonymous questionnaires [16-18,22,23]. Although a potential non-response bias was not formally tested, a higher response rate should reduce the non-response bias [25]; however, a non-response bias might still exist.

Information from 557 farmers was not included in the final model due to non-responses (missing) of one or more questions. There were from 10% to 20% non-responses for each of the questions concerning the farmers' anticipated reactions if scrapie was detected in their flock, which could indicate a non-response bias. We received no indications that these questions were controversial for the farmer, but the farmer might have considered these questions difficult due to their hypothetical form. The non-responses did not show any significantly different distribution for any of the response variables. Therefore, we do not expect that any substantial problem with non-response bias was created by this.

The farmers' awareness of scrapie and their willingness to report scrapie should not be considered static. After this study was conducted, scrapie Nor98 – an atypical scrapie – has constituted most of the scrapie cases detected in Norway [26]. The control measures of scrapie Nor98 have been considered too strict [8] considering the low transmissibility of the disease [26]. The farmers' motivation to report scrapie might therefore have been reduced and hence lead to reduced reporting of scrapie.

## Conclusion

The farmers' vigilance is essential for the detection of classical scrapie as, despite the extensive surveillance programmes, most flocks with classical scrapie have been detected due to cases being notified or animals submitted to the laboratory for disease examination [19]. The results of this study indicate that in 2002 almost all sheep farmers showed willingness to report any scrapie suspects. Nevertheless there is an underreporting of scrapie suspects and the farmers' awareness and hence their vigilance of scrapie could be improved. Consequently, it will be important to continue to motivate the farmers to report, to improve their awareness of scrapie, and to communicate the importance of also reporting animals showing only vague signs or signs typical of differential diagnoses for scrapie. Furthermore, the results suggest that to ensure the farmers' compliance to control programmes for serious infectious diseases, the farmers' concerns of non-economic as well as economic values should be considered.

## Methods

### Design and study population

The study was designed as a cross-sectional study. Three thousand sheep farmers were randomly selected from the Register of Production Subsidies using a pseudo random number procedure [27]. To exclude hobby farmers, only sheep farmers who had ten or more breeding sheep were considered for selection. Altogether 18,404 sheep farmers were eligible.

### Data collection

The data were collected by an anonymous questionnaire survey. The four-page questionnaire contained questions concerning location and flock characteristics, contact patterns and management, and the farmer's knowledge of scrapie and his attitude towards the Norwegian scrapie surveillance and control programme. The questionnaire (in Norwegian) is available from the corresponding author.

The questionnaire was accompanied by a covering letter and a free-post return envelope. It was mailed to all participants in March 2002 followed by a reminder card in April 2002. The data from the questionnaires were entered into an MS access database (Microsoft^® ^Access 2000 Version 9. Microsoft Corporation, WA, USA, 1992–2001).

### The farmers' willingness to report scrapie

The farmers were asked how they would act if they discovered an animal showing signs suggestive of scrapie. Those answering "Report to DVO" or "Submit the material to a diagnostic laboratory" were considered to be "Willing to report scrapie suspects", while those only marking "Send to slaughter" or "Cull the animal" were considered not willing to report these cases. The answer "Don't know" were treated as missing.

Furthermore, the farmers were asked for their reporting behaviour in the situation where an animal had shown clinical signs of listeriosis and did not recover after treatment (hereafter "non-recovering listeriosis cases"). The answers "Contact DVO for examination regarding scrapie" (in short "Notifying") and "Contact veterinary practitioner for further examination" (in short "Re-examine") were kept as separate categories, while the answers "Send to slaughter" or "Kill the animal" were grouped together in the category "Not report" giving three different categories in total. The answer "Don't know" was treated as missing.

### Explanatory variables

#### Demographic information

The farms were located in four geographical regions: South-Eastern, Western, Middle and Northern Norway, based on county. The farmers were asked to specify the flock size within one of four groups: 1–10, 10–49, 50–99 or ≥ 100 breeding sheep.

#### The farmers' knowledge of scrapie signs

Using check-boxes, the farmers were asked to mark the clinical signs they considered as associated with scrapie among ten different clinical signs, where emaciation, hair loss, lip smacking, pruritus and trembling were considered as scrapie-associated signs, and abortion, coughing, diarrhoea, fever, and frequent urinating were considered as not associated with scrapie. The question was considered answered when at least one of the check-boxes was marked. The information was collated into one variable by summing the number of correctly checked scrapie-associated signs. In the multivariate analysis, the variable was treated as a numerical variable.

#### The farmers' anticipated reactions if scrapie should be detected

The information that the detection of a scrapie-positive animal would lead to the destruction of all the sheep in the flock was presented in the questionnaire. The farmers were then asked to give their anticipated reaction to seven concerns regarding the potential detection of scrapie in their flock (Table 1). The reactions were graded in four levels: not important, of minor importance, important, and very important. For the multivariate analysis, each variable was dichotomised to derive two evenly-sized groups (Table 1).

#### The farmers' opinion on factors potentially important for reporting behaviour

The farmers were asked about the importance of four factors which might affect whether they would report an animal with signs suggestive of scrapie. These were: "I need more knowledge of scrapie symptoms", "Having easy access to a DVO", "Being offered free examination of scrapie suspects", and "The Government compensates for the cost of the control measures when scrapie is detected". The answers were graded in four levels: not important, of minor importance, important, and very important. For the multivariate analysis, each variable was dichotomised to derive two evenly-sized groups (Table 1).

### Statistical analyses

The farmers' reporting of non-recovering listeriosis was used as a measurement of the farmers' vigilance in reporting scrapie. The reporting behaviour of non-recovering listeriosis was analysed in a multinomial logistic regression with the farmer as the statistical unit and the response variable categorized in the three nominal categories: Notifying, Re-examine and Not report.

The explanatory variables region, flock size, knowledge of scrapie-associated signs, each of the seven anticipated concerns of the farmer if scrapie were to be detected in their flock, and each of the four factors of potential importance for the reporting of scrapie suspects were considered as candidates for the multivariate analysis. All candidate variables were included in the initial model. Maximum likelihood estimation was used for model fitting, and the likelihood ratio test was used to assess the overall significance of the model. The best model fit was found by using backward stepwise deletion of insignificant terms. For each step the single least explanatory variable was removed until there were no significant difference between the full and all possible reduced models when using the likelihood ratio test with significance level of 0.05. Thereafter, all two-way interaction terms between the remaining explanatory variables were tested one by one for significance in the final multivariate model.

The adjusted RRR (corresponding to the adjusted Odds ratio estimated in binomial logistic regression) were used as measures of association between the response variable and the explanatory variable [28]. For each explanatory variable a separate estimate of the RRR was given for the responses "Notifying" and "Re-examine" relative to the category "Not report" (reference group). For completeness, the RRR for "Notifying" relative to "Re-examine" was calculated from the two other contrasts.

All variable processing and statistical analyses were performed in SAS-PC System^® ^for Windows (SAS Institute Inc., Cary, NC, USA). The descriptive analyses were conducted by using PROC FREQ and missing observations were included when calculating the population percentages. The multinomial logistic regression was conducted by using PROC CATMOD.

## Authors' contributions

PH were main responsible for the design of the study, collecting the data, performing the statistical analysis and drafted the manuscript. SV participated in the design of the study and helped to draft the manuscript. JJ supervised in the design, the statistical analysis and the writing of the manuscript. All authors read and approved the final manuscript.
